# Exposure to Paper Mill Effluent at a Site in North Central Florida Elicits Molecular-Level Changes in Gene Expression Indicative of Progesterone and Androgen Exposure

**DOI:** 10.1371/journal.pone.0106644

**Published:** 2014-09-08

**Authors:** Erica K. Brockmeier, B. Sumith Jayasinghe, William E. Pine, Krystan A. Wilkinson, Nancy D. Denslow

**Affiliations:** 1 Department of Physiological Sciences, Center for Environmental and Human Toxicology, University of Florida, Gainesville, Florida, United States of America; 2 Department of Wildlife Ecology and Conservation, University of Florida, Gainesville, Florida, United States of America; 3 Chicago Zoological Society, c/o Mote Marine Laboratory, Sarasota, Florida, United States of America; 4 Genetics Institute, University of Florida, Gainesville, Florida, United States of America; University of North Carolina at Chapel Hill, United States of America

## Abstract

Endocrine disrupting compounds (EDCs) are chemicals that negatively impact endocrine system function, with effluent from paper mills one example of this class of chemicals. In Florida, female Eastern mosquitofish (*Gambusia holbrooki*) have been observed with male secondary sexual characteristics at three paper mill-impacted sites, indicative of EDC exposure, and are still found at one site on the Fenholloway River. The potential impacts that paper mill effluent exposure has on the *G. holbrooki* endocrine system and the stream ecosystem are unknown. The objective of this study was to use gene expression analysis to determine if exposure to an androgen receptor agonist was occurring and to couple this analysis with *in vitro* assays to evaluate the presence of androgen and progesterone receptor active chemicals in the Fenholloway River. Focused gene expression analyses of masculinized *G. holbrooki* from downstream of the Fenholloway River paper mill were indicative of androgen exposure, while genes related to reproduction indicated potential progesterone exposure. Hepatic microarray analysis revealed an increase in the expression of metabolic genes in Fenholloway River fish, with similarities in genes and biological processes compared to *G. holbrooki* exposed to androgens. Water samples collected downstream of the paper mill and at a reference site indicated that progesterone and androgen receptor active chemicals were present at both sites, which corroborates previous chemical analyses. Results indicate that *G. holbrooki* downstream of the Fenholloway River paper mill are impacted by a mixture of both androgens and progesterones. This research provides data on the mechanisms of how paper mill effluents in Florida are acting as endocrine disruptors.

## Introduction

In the early 1980s, female Eastern mosquitofish (*Gambusia holbrooki*) with elongated anal fins, mimicking the fins of adult males, were first documented from Elevenmile Creek, Florida [1]. This small stream served as the wastewater outfall of a large paper mill for several decades and has been known to be contaminated both “chemically and biologically” [2]. Additional surveys for masculinized female *G. holbrooki* found similar results downstream of other paper mill impacted sites in Florida including the St. John's River at Rice Creek [3] and the Fenholloway River [4]. These findings are not limited to the US, as masculinized female Western mosquitofish (*G. affinis*) have recently been found downstream of an active paper mill in China [5]. Regulatory policies in the US such as the Clean Water Act have led to improvements in toxicity of industrial effluents via reductions of chemicals such as dioxins, ammonia, and metals. However, endocrine disrupting chemicals (EDCs) are often still prevalent in this type of effluent, with aquatic wildlife residing downstream of paper mill-impacted areas exhibiting increased proportions of single sex embryos, abnormal sex steroid levels, modulations in egg yolk proteins, and reduced gonad sizes [6].

Over the last decade, numerous research efforts have been conducted to assess the causes of masculinization of *G. holbrooki* in the Fenholloway River [7]–[12]. Previous high performance liquid chromatography (HPLC) fractionation studies determined that chemicals in water samples from this site could bind the human androgen receptor (AR) and induce androgen-dependent gene expression [7]. The compound androstenedione (AED), a precursor of testosterone in the steroidogenic pathway, was initially associated with the activation of the AR, and this chemical was found in Fenholloway River water and sediment. However, follow-up analysis demonstrated that AED was not in the individual HPLC fraction associated with the AR activation, as fractions were pooled before chemical identification was conducted [8], [9]. Furthermore, a 6-week exposure of *G. affinis* to the concentration of AED present in Fenholloway River water (0.04 ng/L) did not result in anal fin masculinization [10].

Other biological endpoints in *G. holbrooki* from the Fenholloway River suggest larger liver masses and smaller body masses and lengths, as well as an increase in the number of oocytes in masculinized female *G. holbrooki* from the Fenholloway River [11]. Later studies found reductions in the number of early and late stage embryos in *G. holbrooki* residing downstream of the paper mill [12], potentially due to the lack of normalization of these endpoints to body mass [11], [12].

Previous studies in the Fenholloway River succeeded in evaluating the physiological impacts of paper mill exposure on resident mosquitofish. However, better insights into the mechanisms of action of chemicals in paper mill effluent can be obtained by including molecular endpoints in these analyses [13]. mRNA levels of bone growth factor *sonic hedgehog* (*shh*) [14], [15] and steroidogenic enzyme *17β-hydroxysteroid dehydrogenase 3* (*17βhsd3*) are potential biomarkers of androgen exposure in *G. holbrooki* [16], [17]. In fish, *vitellogenin* (*vtg*) and *zona pellucida* glycoproteins (*zp*) are crucial components of oocyte quality [18] and decreases in their expression often occur during androgen exposure [15], [19], which can possibly lead to low-quality eggs. Hepatic transcriptome analysis can provide mechanistic information on the impacts of toxicant exposure, as the liver is the primary detoxification organ and comes into contact with any chemicals that fish are exposed to in the field.

Transcriptomics experiments of fish exposed to androgenic chemicals have revealed large impacts on the biological processes of metabolism [16], [19] so focusing on the liver is of relevance for evaluating the androgenicity of paper mill effluents. In addition, *in vitro* methods in the lab can be used to help screen samples of unknown chemical contaminants for their ability to activate the transcription of genes via nuclear receptors. This has been previously demonstrated in the Fenholloway River below an active paper mill [7] and when data from these assays are coupled with molecular data from the fish impacted by the effluents, they can provide additional information for determining the type(s) of chemicals present and impacting aquatic organisms at this location.

The objective of this study was to evaluate changes in gene expression coupled with *in vitro* nuclear receptor assays to evaluate the androgenicity of water downstream of the paper mill on the Fenholloway River. Two specific aims were developed: (1) evaluate mRNA levels of *vtg*, *17βhsd*3, and *zp2* in the liver, *shh* in the anal fin, and global hepatic gene expression profiles associated with paper mill exposure, and (2) determine if chemicals in the Fenholloway River could bind to the ligand binding domain of androgen and progesterone receptors. We hypothesized that modulations in gene expression patterns and *in vitro* analyses would be indicative of androgen exposure and that global gene expression analysis via microarrays would provide insights into the mode(s) of actions of the chemicals present in the effluent. This addresses an important gap in knowledge by evaluating the type(s) of EDC that impact *G. holbrooki* at this site.

## Materials and Methods

### Sample collection

**Figure 1:**
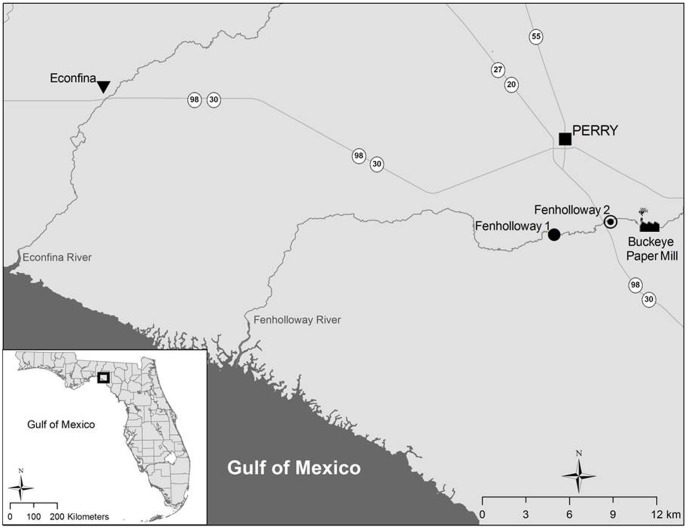
Spatial representation of the two sites where female Eastern mosquitofish (*Gambusia holbrooki*) were collected for this study. The paper mill-impacted sites include Fenholloway River sites 1 and 2 and the reference site in the Econfina River. *G. holbrooki* were collected from Fenholloway site 1 on July 12^th^, 2012 and from Fenholloway site 2 on April 10^th^, 2013. *G. holbrooki* were collected from the Econfina River on August 7^th^ and October 4^th^, 2012. The map was created using ArcGIS v10.0 (Esri, Redlands, USA).

Wild *G. holbrooki* were captured from two sites downstream of the Buckeye Kraft Pulp and Paper Mill on the Fenholloway River as well as one site at the Econfina River which does not receive paper mill effluent and served as a reference site (Fig. 1). Only sexually mature *G. holbrooki* (females >15 cm standard length and with the presence of the gravid spot) were collected. In Summer 2012, three sampling events were conducted, once at the Fenholloway River downstream of the paper mill along County Road 361A (Fenholloway 1; GPS coordinates: N 30 058.341′, W 83 588.569′) and twice on the Econfina River (GPS coordinates: N 30 08.549′, W 83 51.962′). Additional sampling was conducted in 2013 to obtain fin samples from the Fenholloway River along US 98 (Fenholloway 2; GPS coordinates N 30 03.925′, W 83 33.470′) and the Econfina River. A 1/8″ mesh seine was used to collect *G. holbrooki*. This study was carried out in accordance with the recommendations laid out in “Guide for the Care and Use of Laboratory Animals” by the National Institutes of Health. The protocol was approved by the Institutional Animal Care and Use Committee of the University of Florida (Protocol 201105665). Field sampling was conducted under Florida Fish and Wildlife Commission fishing licenses; all water access points were public areas and no protected species were sampled.

Fish were transferred to 5 gallon aerated buckets filled with site water and were processed immediately after collection. Fish were euthanized using a lethal dose (300 mg/L) of Tricaine-S (Western Chemical, Ferndale, USA) and sacrificed via spinal transection. Anal fin elongation and oocyte stage, staged according to previously published criteria, [20] were assessed upon dissection. Livers and anal fins were excised and stored in RNAlater (Qiagen, Hilden, Germany) overnight at 4°C before long-term storage at −80°C. A subset of fin samples collected in 2012 (N = 4 per site) was used for evaluating the number of bone segments in the third ray of the anal fin using methods previously described [15].

For water samples, collection took place at each of the three sites previously indicated as well as at a site upstream of the Econfina River collection area (GPS coordinates N 30 08.549′, W 83 51.962′) in April 2013. EPA-approved 2.5 L amber bottles (Fisher Scientific, Hampton, USA) were filled with site water by placing the open beaker into the river using a 3 meter long net with only flowing surface water collected. Water was kept on ice immediately after collection, brought to pH<4 to prevent bacterial growth after returning to the laboratory, and stored at 4°C until further processing.

### RNA extraction

RNA was extracted from livers by phenol-chloroform extraction using methods previously described [15]. For anal fin RNA extraction, samples stored in RNAlater were blotted dry before purification using the RNeasy kit (Qiagen, Hilden, Germany) following the manufacturer's protocol. Fins were homogenized in 600 µL lysis buffer (Buffer RLT; Qiagen, Hilden, Germany) for 20 seconds and centrifuged for 3 minutes at 4°C at 20,800 g. The supernatant was mixed with 600 µL 70% (v/v) ethanol, transferred to an RNeasy column, and centrifuged for 15 seconds at 4°C at 20,800 g. Samples were then washed with 350 µL wash buffer (Buffer RW1; Qiagen, Hilden, Germany) for 15 seconds at 4°C at 20,800 g and an on-column DNase treatment (RNA-free DNase kit, Qiagen, Hilden, Germany) was conducted for 15 minutes at room temperature before washing and centrifuging with 350 µL Buffer RW1 for 15 seconds at 4°C at 20,800 g. Two washes of 500 µL concentrated wash buffer (Buffer RPE; Qiagen, Hilden, Germany) for 15 seconds and 2 minutes at 4°C at 20,800 g were conducted before elution in 30 µL RNase-free water. RNA quality and quantity of all samples were assessed using the Nanodrop (ThermoScientific, Waltham, USA). The A_260_/A_280_ values of all samples ranged from 1.75 to 2.18.

### Quantitative polymerase chain reaction analysis

Real-time quantitative polymerase chain reaction (qPCR) analysis was performed using the myiQ single color real-time PCR detection system and analyzed by the iQ software (BioRad, Hercules, USA) as previously described [15]. 200 ng cDNA from livers and 25 ng cDNA from anal fins was amplified using SYBR Green (BioRad, Hercules, USA). Negative controls of water (no template control) and RNA (minus RT control) were analyzed on each plate. Gene copy number and qPCR efficiencies for all genes except *shh* were determined using absolute standard curves [15]. For *shh*, serial dilutions of anal fin RNA were used to calculate reaction efficiency. The average qPCR reaction efficiency was 93.6%. Melt curve analysis revealed that all samples formed a single peak, with no primer dimers or alternate products formed. *RPL8* variability across all samples was 9.67%. qPCR primer sequences can be found in the supporting information (Table S1 in File S1).

### Microarray sample preparation

RNA integrity numbers (RINs) of stage-matched RNA samples (N = 4 per site) were determined with the 2100 BioAnalyzer (Agilent, Santa Clara, USA); samples had a range of RIN's from 7.8 to 8.9. The Low RNA Input Amplification Kit for one color labeling (Cy3) (Agilent, Santa Clara, USA) was used to synthesize cRNA using procedures previously described [16]. Purified cRNA concentrations and specific activities were determined by Nanodrop (ThermoScientific, Waltham, USA) with a specific activity >8 as the cut-off for further processing [16]. Hybridization of 600 ng cRNA and slide scanning was conducted as previously described [16]. The microarray data set (GSE49238) was submitted to the Gene Expression Omnibus (GEO) database, with all reports and deposits made according to the Minimum Information About a Microarray Experiment (MIAME) guidelines [21].

### Water sample extraction and *in vitro* analysis

Water (1.5 L) from each of the four collection sites was passed through Whatman filter paper (GE Healthcare, Maidstone, UK) via vacuum filtration and stored at 4°C until solid phase extraction (SPE). Water samples were extracted using 6 mL Oasis HLB columns (Waters, Milford, USA) after conditioning the columns with 5 mL of methanol and 5 mL of milli-Q water. Water samples were passed through columns using a vacuum manifold with a flow rate of 10 mL/min. After the entire sample was passed through the cartridge, organic constituents were eluted with 5 mL of methanol, evaporated to dryness with nitrogen, reconstituted in 100 µL of dimethyl sulfoxide (DMSO), and stored at −80°C until bioanalysis.

The GeneBLAzer progesterone receptor (PR) and androgen receptor (AR) assays (Life Technologies, Carslbad, USA) were conducted as previously described [22]. This assay functions in stably transfected mammalian cells with a chimera receptor gene that contains the human AR or PR ligand binding domain linked to the DNA binding domain of Gal 4. The cells also contain a stably transfected reporter gene with the DNA binding sites for Gal 4 in the promoter region upstream of the beta-lactamase gene. In the presence of ligands for the receptors, the chimera is transactivated and binds to sites on the promoter for the beta lactamase gene, resulting in transcription and translation of the beta lactamase enzyme. To measure beta lactamase activity, a substrate with two fluors is used; these fluors are in close proximity and exhibit fluorescence resonance energy transfer (FRET) at 520 nm (green spectrum) and upon hydrolysis the substrate fluoresces at 447 nm (blue spectrum).

To measure the amount of ligand in the water extract, the DMSO reconstituted samples were diluted 1∶200, 1∶400, 1∶800 and 1∶1600 in cell culture media. After an 16 h incubation, cells were loaded with LiveBLAzer-FRET B/G Substrate and incubated for 2 h. Fluorescence emission values at 460 nm and 530 nm were obtained using a standard fluorescence plate reader (Synergy H1 Hybrid Reader, Bio-Tek, Winooski, USA). The samples were analyzed on the same plate as a standard curve of levonorgestrel and R1881 for the PR and AR assays respectively. Bioanalytical equivalence quotients (BEQs) were calculated for the field samples by comparing the activity of the water samples to the standard curves following previously described methods [22] (Fig S3 in File S1). The EC_10_ (10% effect concentration) value was used and extrapolated back to the original 1.5 L of water collected at the sites to determine the chemical concentrations at each site. Data is presented as a ratio of blue spectrum values to green spectrum value, which is indicative of PR or AR transactivation.

### Statistical analysis

Anal fin elongation was quantified by methods previously described [15] as a ratio of the measurement of the base of the anal fin (ray 6) to the total length of the fin (ray 4). Liver qPCR data were analyzed as the ratio of the gene copy number per ng RNA versus the *RPL8* copy number per ng RNA multiplied by the average *RPL8* value. All hepatic gene expression results were log-transformed before statistical analyses. Statistical analysis of *shh* was conducted by the delta delta Ct method as previously described [16]. A Student's t-test was used (after confirming normality and homogeneity of data distributions) to determine if there was a statistically significant difference between anal fin elongation, bone segment numbers, and gene expression levels between the Fenholloway River and the Econfina River. Results of all statistical tests were considered significant at α = 0.05.

Microarray data quality control analysis was completed as described previously [16] and data processing and analysis were conducted with JMP Genomics 6.0 (SAS Institute, Cary, USA). A one-way analysis of variance (ANOVA) was conducted after LOcal regrESSion (loess) normalization to determine genes that were differentially expressed between sites. Genes that were statistically significantly differentially expressed with >1.5-fold change were subjected to hierarchical cluster analysis [16], [22], [23]. Changes in Gene Ontology (GO) Biological Processes were determined using the Fisher's Exact test (Fisher raw p-value <0.05, FDR α = 0.05). Visualization of significant pathways and genes was conducted using PathwayStudio (Elsevier, Amsterdam, The Netherlands) based on ResNet 9.0.

Gene expression similarities were evaluated between female *G. holbrooki* downstream of the paper mill-impacted site and *G. holbrooki* that were exposed to the potent androgen 17β-trenbolone (TB) [16]. For this analysis, a one-way ANOVA was used to determine genes that were differentially expressed between samples from the Fenholloway River, the Econfina River, and the TB exposure versus the vehicle control. Only mRNAs that were altered in a statistically significant manner in any group (paper mill collection sites or laboratory TB treatment) compared to controls were used in further analysis. This focused list of genes from this set that were expressed in the same direction in the paper mill-impacted site fish and the TB-exposed fish were subjected to cluster analysis [16], [23], [24].

## Results

### Field sites and anal fin elongation

**Figure 2:**
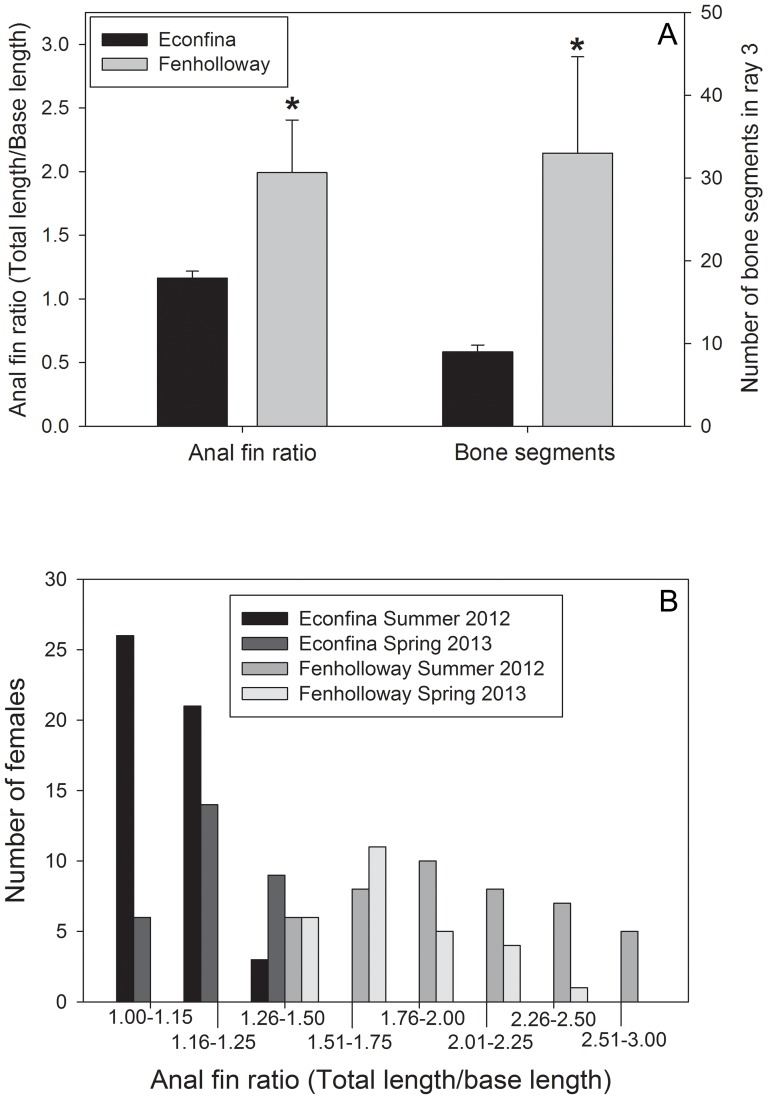
Anal fin elongation and bone segment formation in anal fin ray 3 of female Eastern mosquitofish (*Gambusia holbrooki*) collected from the Fenholloway and Econfina Rivers. Data are repsresented as (A) Mean (± standard deviation) of all collection events and, (B) Distribution of anal fin elongation classes between both paper mill exposed (Fenholloway) and reference (Econfina) field sites. An asterisk indicates statistical significance between the two groups as determined using a Student's t-test with p<0.05. For anal fin elongation levels there was an N of 46 and 50 from the Fenholloway and Econfina rivers respectively and a subset N of 4 from both sites for the bone segment evaluation.

Since the Econfina River has been used in previous studies as a control site for the Fenholloway River [7], [11], [12], [25] we selected the Econfina as the reference site due to the foundation of knowledge available for this location. The selection of a reference site in a separate system was further justified due to the reduced potential for complications from upstream migration of paper mill-impacted fish. A significant increase in anal fin elongation was seen in *G. holbrooki* from the Fenholloway River (Student's t-test, p<0.001) as well as the number of bone segments in the third ray of the anal fin (Student's t-test, p = 0.029) (Fig. 2A) which was absent in females from the Econfina River. The distribution of anal fin elongation size classes based on site and collection season is also provided, demonstrating an increase in the number of masculinized females in the Fenholloway River during both collection seasons (Fig. 2B).

### Focused Gene Expression Analyses

**Figure 3:**
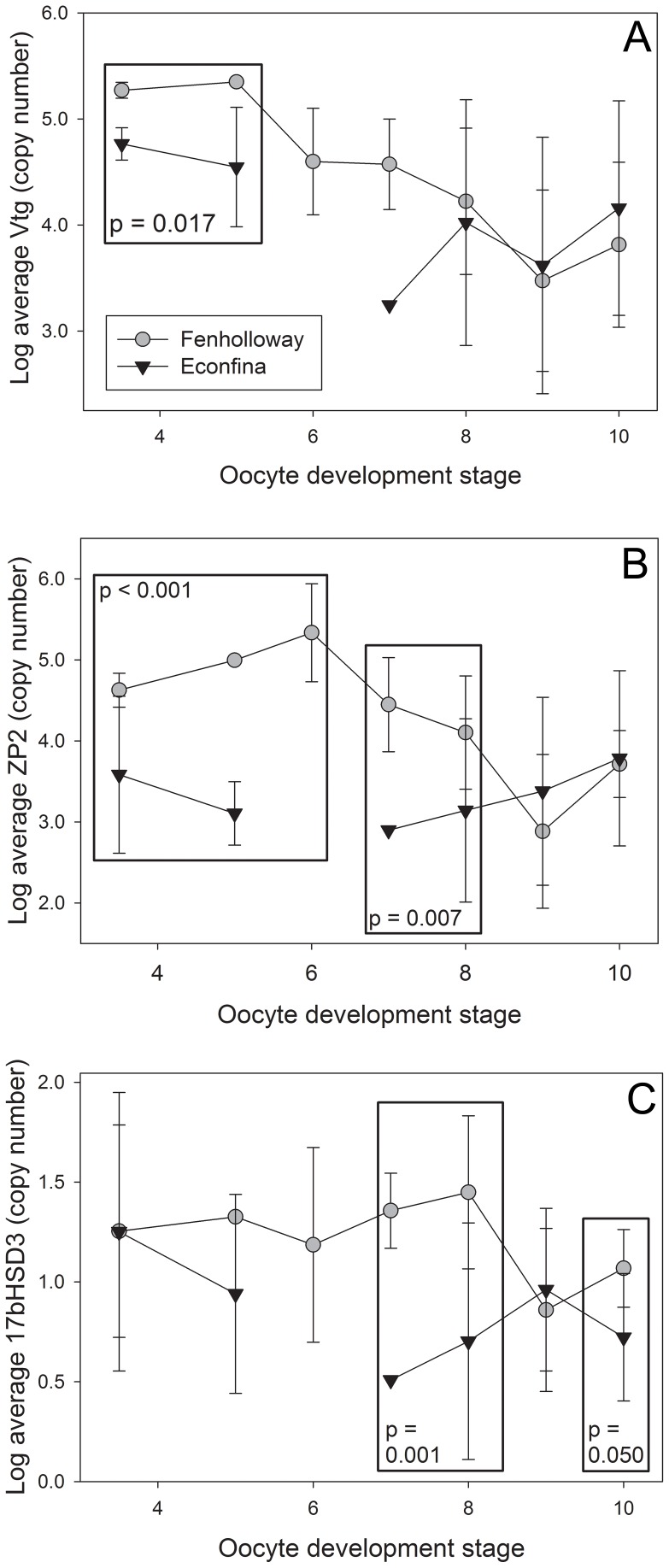
Focused hepatic gene expression patterns in female Eastern mosquitofish (*Gambusia holbrooki*) using qualitative polymerase chain reaction analysis. Gene expression of (A) vitellogenin (*vtg*), (B) zona pellucida glycoprotein 2 (*zp2*), (C) 17β-hydroxysteroid dehydrogenase 3 (*17βhsd3*) was analyzed between paper mill exposed (Fenholloway) and reference (Econfina) collection sites. Each point represents the mean of each group and error bars represent standard deviation; time points with no standard deviation have an N of 1. A box indicates statistical significance between groups at the selected oocyte developmental stages as determined using a Student's t-test with p<0.05.

**Figure 4:**
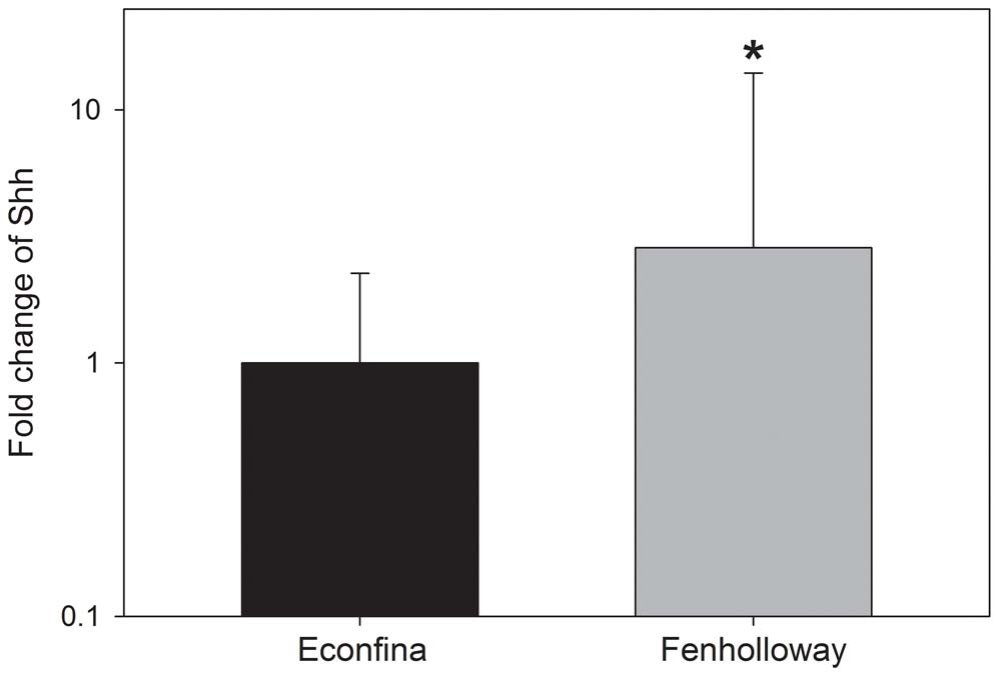
Anal fin gene expression patterns of sonic hedgehog (*shh*) in female Eastern mosquitofish (*G. holbrooki*). An asterisk indicates statistical significance between the paper mill exposed (Fenholloway) and reference (Econfina) rivers as determined using a Student's t-test with p<0.05.

An increase in the mRNA levels of hepatic *vtg*, *zp2*, and *17βhsd3* was seen in female *G. holbrooki* from the Fenholloway River (Fig. 3). Due to differences in gene expression based on oocyte development stage [26], a *post-hoc* analysis was conducted. For *vtg*, significantly increased expression was observed at early oocyte development stages (Student's t-test, p = 0.017 stages 3 through 5) (Fig. 3A), for *zp2* at both early and mid-stages of development (Student's t-test, p<0.001 stages 3 through 5; p = 0.007 stages 7 through 8), (Fig. 3B), and for *17βhsd3* at mid-stages of oocyte development (Student's t-test, p = 0.001 stages 7 through 8; p = 0.050 stage 10) (Fig. 3C). There was also significantly increased expression of *shh* in masculinized Fenholloway River female *G. holbrooki* anal fins (Student's t-test, p = 0.012, N = 22 Econfina River and 16 Fenholloway River) (Fig. 4).

### Hepatic Transcriptome and Pathway Analysis

Hepatic gene expression analysis revealed significant differential gene expression patterns between the Fenholloway River and Econfina River *G. holbrooki*, with 121 genes upregulated and 91 genes downregulated in the Fenholloway River (One-way ANOVA, p<0.05, FDR α = 0.05, fold change >±1.5) (Fig. S1 in File S1). A complete list of all statistically significant transcripts with >±1.5 fold change can be found in the supporting information (File S2).

In total, 22 gene ontology (GO) biological processes were significantly enriched in *G. holbrooki* from the Fenholloway River (Fisher's Exact test, p<0.05) (Table 1). Of note are pathways related to metabolism (isoprenoid biosynthetic process, energy reserve metabolic process, cyclic nucleotide biosynthetic process), reproduction (gonad development, cell migration involved in gastrulation), and nuclear processes (rRNA processing, mRNA transport, DNA topological change). *G. holbrooki* exposed to the potent androgen receptor agonist 17β-trenbolone (TB) in the lab [16] and *G. holbrooki* from the Fenholloway River share some similarly enriched GO biological processes, including regulation of protein metabolic process (go:0051246) and mRNA transport (go:0051028). Enriched processes and their connection to genes were visualized by PathwayStudio (Fig. S2 in File S1) and these results corroborate the findings of the Fisher's Exact test while revealing interactions between key metabolic pathways and the differential regulation of several genes linked to these processes.

**Table 1:** Significantly differentially regulated biological processes in female *G. holbrooki* residing downstream of a paper mill as compared to a control site as determined by gene set enrichment and gene ontology (GO) analyses.

Gene Ontology Biological Process	Fisher's Raw p-value	Type of regulation	Dif. reg^a^	Not dif. reg^b^
go:0006511; ubiquitin-dependent protein catabolic process	0.00659	Enriched	0.950%	0.472%
go:0006816; calcium ion transport	0.00968	Enriched	0.712%	0.328%
go:0051603; proteolysis involved in cellular protein catabolic processes	0.00014	Enriched	0.626%	0.144%
go:0006364; rrna processing	0.02156	Enriched	0.561%	0.246%
go:0051246; regulation of protein metabolic process	0.02663	Enriched	0.453%	0.185%
go:0044419; interspecies interaction between organisms	0.01696	Enriched	0.410%	0.144%
go:0001947; heart looping	0.04791	Enriched	0.388%	0.164%
go:0051028; mrna transport	0.00914	Enriched	0.367%	0.103%
go:0009615; response to virus	0.04432	Enriched	0.259%	0.082%
go:0042074; cell migration involved in gastrulation	0.01105	Enriched	0.237%	0.041%
go:0008299; isoprenoid biosynthetic process	0.00075	Enriched	0.216%	0.00%
go:0008045; motor axon guidance	0.00526	Enriched	0.216%	0.021%
go:0018149; peptide cross-linking	0.01959	Enriched	0.216%	0.041%
go:0009887; organ morphogenesis	0.00994	Enriched	0.194%	0.021%
go:0006890; retrogrAED vesicle-mediated transport, golgi to er	0.00318	Enriched	0.173%	0.000%
go:0048738; cardiac muscle tissue development	0.01865	Enriched	0.173%	0.021%
go:0008406; gonad development	0.00652	Enriched	0.151%	0.000%
go:0006265; dna topological change	0.03469	Enriched	0.151%	0.021%
go:0007266; rho protein signal transduction	0.03469	Enriched	0.151%	0.021%
go:0006112; energy reserve metabolic process	0.02748	Enriched	0.108%	0.000%
go:0009190; cyclic nucleotide biosynthetic process	0.02748	Enriched	0.108%	0.000%
go:0016339; calcium-dependent cell-cell adhesion	0.02748	Enriched	0.108%	0.000%

a Percentage of genes within the GO category that were significantly differentially regulated between Econfina and Fenholloway groups.

b Percentage of genes within the GO category that were not significantly differentially regulated between Econfina and Fenholloway groups.

**Figure 5:**
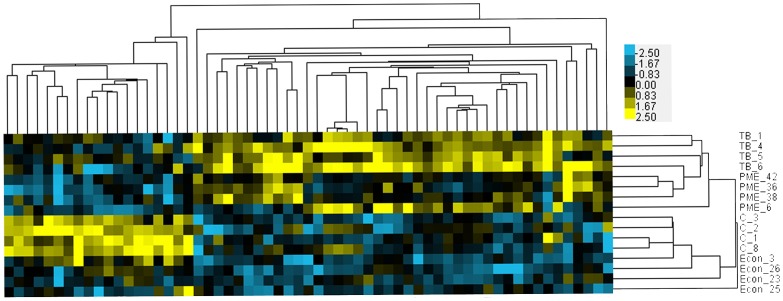
Gene expression profile comparisons between Fenholloway (PME) and reference site (Econ) female *G. holbrooki* and female *G. holbrooki* exposed to the androgen 17β-trenbolone (TB) or the vehicle control (C). This set of genes were differentially expressed between the PME, Econfina, and TB groups over the lab controls with at least a 1.5-fold difference of expression over the lab control and was expressed in the same direction in those groups. Data were median-centered by gene and clustered using spearman correlation and centroid linkage. Yellow genes are more highly expressed than the gene average and blue genes are expressed at a lower level than the gene average.

We found similarity between hepatic gene expression data sets in *G. holbrooki* from the paper mill-impacted site and *G. holbrooki* exposed to 1 µg TB/L for 14 days when compared to those from the reference site or exposed to a vehicle control (Fig. 5) [16]. In a set of 62 similarly regulated genes, TB-exposed and PME-exposed *G. holbrooki* hepatic expression profiles cluster together, and the reference site and vehicle control samples cluster separately (Fig. 5). A complete list of these genes can be found in the supporting materials (File S3).

### *In vitro* assay results

**Figure 6:**
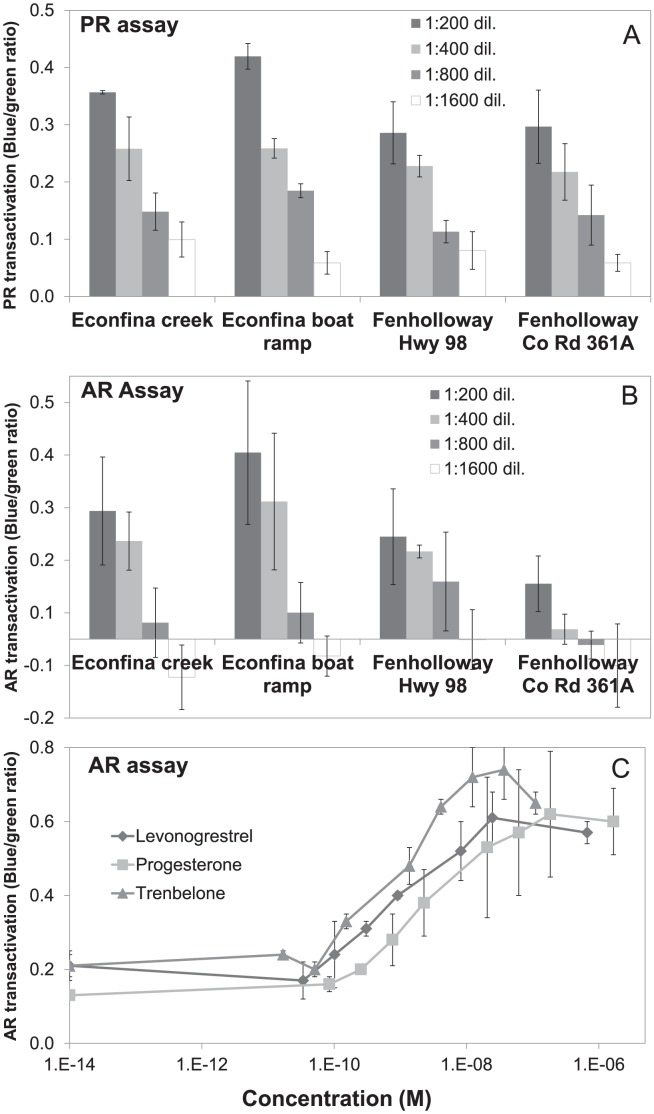
Results of progesterone receptor (PR) and androgen receptor (AR) GeneBLAzer assays for concentrated water samples collected downstream of the Fenholloway River paper mill and the Econfina River conservation area. The graph bars represent the mean of three replicates from each dilution for the PR (A) and AR (B) assays. Error bars represent standard deviation of the three replicates per assay. The dose-responses of levonorgestrel, progesterone, and 17β-trenbolone (C) were also evaluated by the AR assay.

Fig. 6A demonstrates the PR ligand transactivation of chemicals present in water collected from both the reference and paper mill impacted field sites. All water samples had detectable levels of PR activation as determined by the GeneBLAzer assay. The levonorgestrel BEQs of the field site water are as follows: Econfina creek, 3.33 ng/L (10.65 pM); Econfina boat ramp, 4.24 ng/L (13.56 pM); Fenholloway Hwy 98, 3.21 ng/L (10.28 pM); and Fenholloway Co Rd 361A, 3.28 ng/L (10.48 pM). Both sites also had detectable AR transactivation (Fig. 6B) but at a reduced concentration as compared to the PR assay. The R1881 BEQs of these sites are: Econfina creek, 2.24 ng/L (7.86 pM); Econfina boat ramp, 2.58 ng/L (9.07 pM); Fenholloway Hwy 98, 1.94 ng/L (6.82 pM); and Fenholloway Co Rd 361A, 0.804 ng/L (2.83 pM). Using this assay, levonorgestrel was also demonstrated to activate the AR in the GeneBLAzer assay more strongly than P4 (Fig. 6C).

## Discussion

We found that masculinization of female *G. holbrooki* continues to occur in the Fenholloway River. Paper mill effluent exposure is associated with both anal fin elongation as well as with significantly increased bone segment formation at this site. Additionally, we found an increase in the mRNA levels of *vtg, zp2, 17βhsd3*, and *shh* in Fenholloway River *G. holbrooki*. Through comparison of hepatic gene expression patterns to data from laboratory exposures, we found that paper mill effluent exposure resulted in an increase of genes associated with metabolic pathways, with 62 genes similarly expressed by *G. holbrooki* exposed to androgens, indicating a similarity between impacts at the molecular level between paper mill and androgen exposure. We also found detectable levels of both AR and PR ligands in the transactivation assay in concentrated water samples collected from both the paper mill impacted and reference sites.

Anal fin ratios correlate with earlier observations of elongation in field samples collected in 2010 (Brockmeier, unpublished results) and the increase in the level of bone segmentation is also similar to previous findings [7], [12]. Because the mechanism of anal fin growth in mosquitofish is associated with androgen concentration [14], [27], [28], these data appear to support an ongoing androgen exposure occurring at this site. In addition to anal fin growth, the mRNA levels of *shh* were upregulated in females from the Fenholloway River (Fig. 4). This gene is expressed in mosquitofish fry and adult females during androgen exposure [14], [15], and is constitutively expressed in adult male anal fins (Erica K. Brockmeier, Ph.D. dissertation, University of Florida, 2013). Other androgenic gene expression responses have also been seen downstream of other paper mills, such as *spiggin* expression in the kidneys of female three-spined stickleback (*Gasterosteus aculeatus*) exposed to paper mill effluent [29]. This gene is, however, only usable in members of the *Gasterosteus* genus, which is not found in Florida. The benefit of measuring *shh* or other biomarker genes to evaluate androgenicity, in addition to anal fin elongation, is that significant changes in gene expression occurs at time points of exposure earlier than physiological responses [14], [15]. This sensitivity in terms of exposure timing can serve as an ‘early warning’ biomarker, indicating exposure to an androgenic compound before the physiological effects of anal fin growth manifest.

We found significant differences in hepatic mRNA expression for *vtg* and *zp2* gene (Fig. 3A and 3B). Differences in *vtg* occurred for oocyte development stages where active vitellogenesis occurs [20] and *zp2* differences were apparent during later stages of oocyte development, corresponding to this gene's role in oocyte maturation [18]. Previous laboratory studies in fish have indicated that exposure to androgenic chemicals negatively impact *vtg* and *zp* gene expression in *G. holbrooki* [15], [16], as well as in other fish species [19], [30]. Our findings of increases in these mRNAs did not support the initial hypothesis. However, our findings match results from other fish species exposed to paper mill effluent extracts. The *Vtg* protein was significantly increased in rainbow trout (*Oncorhynchus mykiss*) after intraperitoneal (IP) injection with primary extracts [31] and after exposure to 100% combined mill outfall (CMO) and in 10% untreated kraft effluent exposures to fathead minnow [32]. While gene and protein levels are not always directly correlated, the upregulation of this protein in rainbow trout and fathead minnow and the gene in mosquitofish may be due to the presence of phytoestrogens in these effluents such as β-sitosterol [6]. This supports the findings of paper mill-specific gene expression patterns, as both androgenic and estrogenic responses were observed.

Hepatic *17βhsd3* mRNA was significantly increased in *G. holbrooki* residing downstream of the pulp and paper mill plant on the Fenholloway River (Fig. 3C). This increase was seen at advanced oocyte development stages [20], [26]. Other forms of *hsd* proteins, including the protein *3βhsd*, have also been found upregulated in the livers of male fathead minnows (*Pimephales promelas*) after exposure to CMO [33]. Previous work conducted at the Fenholloway River indicated that males from both the paper mill impacted and the reference sites had similar levels of both testosterone and E2, as was the case for females [11]. Due to the lack of differences in sex steroid levels among *G. holbrooki* from these sites, it is possible that induced expression of genes for steroidogenic enzymes such as *17βhsd3* may modulate abnormal steroid levels as a homeostatic response to EDC exposure [34]. In addition, other enzymes related to steroid synthesis, such as aromatase, also have significantly increased activities in *G. holbrooki* in the Fenholloway River [25].

A distinct pattern of differential gene expression was present in the livers of female *G. holbrooki* from the Econfina and the Fenholloway rivers (Fig. S1 in File S1). Using gene ontology analysis, numerous pathways and processes were found to be enriched in the livers of the Fenholloway River *G. holbrooki* (Table 1, Fig. S2 in File S1). Notable pathways include nuclear processes such as mRNA transport and rRNA processing as well as several pathways linked to metabolism, including regulation of protein metabolic process, isoprenoid biosynthetic process, and energy reserve metabolic process. Similar results were found in studies conducted on fathead minnows exposed to the CMO of an androgenic paper mill effluent in Canada, another paper mill with processes similar to those used at the Fenholloway River site [33].

In male fathead minnows, the isoprenoid biosynthetic process was also upregulated in the liver, along with several pathways linked to steroid and cholesterol metabolic processes. Female fathead minnows exposed to CMO experienced enrichment in the proteolysis pathway [33], similar to our results of significantly increased ubiquitin-dependent protein catabolic process. Isoprenoid biosynthesis is the necessary precursor for cholesterol synthesis, which serve as templates for sex steroid synthesis. Genes upstream of the synthesis of cholesterol have previously been found to be upregulated by androgen exposure [35], lending support to the hypothesis that chemicals present in paper mill effluents are acting to disrupt the normal metabolism of steroids and hormones in a manner similar to androgen exposures [31].

We found a similarity in hepatic gene expression between TB-exposed *G. holbrooki* and *G. holbrooki* from the Fenholloway River (Fig. 5). Within this list of genes, GO biological processes for metabolic process, biosynthetic process, regulation of transcription, and steroid biosynthetic process are present, providing additional evidence for the importance of changes to metabolic pathways—notably steroid biosynthesis—as the toxic mechanism of action of androgen and paper mill effluent exposure.

Progesterone and androgen receptor active chemicals were found to be present at two sites downstream of the paper mill-impacted area of the Fenholloway and Econfina rivers as determined by the GeneBLAzer assay (Fig. 6). While this is not a direct measurement of chemicals (e.g. GC-MS), we were able to determine a concentration of chemicals which could bind to the ligand binding domain of both the PR and AR and transactivate the receptors. We report their bioanalytical equivalency quotients (BEQs), calculated based on the positive standards R1881 and levonorgestrel as known AR and PR activators respectively. These values were between 3.28 and 4.24 ng/L levonorgestrel equivalencies and between 0.804 and 2.58 ng/L R1881 equivalencies respectively. Based on these results, it appears that natural progesterones are not the causative chemical(s) in inducing abnormal anal fin elongation in the Fenholloway River, as PR-positive activity is present both in the Fenholloway River downstream of the paper mill and at the Econfina River. In previous studies, both progesterone (P4) and the weak androgen androstenedione (AED) were present in HPLC fractions that induced androgen receptor-mediated activity using an *in vitro* assay [7], [9]. AED was found in the water column and sediment of the Fenholloway River at 0.04 µg/L and 0.7 µg/L respectively, whereas P4 was present at much higher levels at 2.06 µg/L (water) and 48.8 µg/L (sediment) [36]. Compared to these concentrations, the effective concentrations found in the water samples in this study are reduced by ∼100-fold and ∼4-fold, as determined by levonorgestrel and R1881 equivalencies respectively (Fig. 6).

The presence of low levels of AR-active chemicals at both sites may be due to the breakdown of this chemical from high levels of progesterones in the sediments via microorganisms [37]. The source of these progesterones is most likely the loblolly pine (*Pinus taeda L.*) which is highly prevalent along this entire area [38]. As anal fin elongation is found only at the Fenholloway River, this suggests that the causative chemical is specific to that site or may be related to a combined effect from both progesterone and androgen loads. While chemicals in the sediment may not normally be available to *G. holbrooki*, as they generally feed off organisms that reside towards the water surface, abiotic pressures present in the Fenholloway River, such as very low dissolved oxygen, may drive *G. holbrooki* in this area to alternative food sources at the river bottom (i.e. Nematocera) [39]. This would result in the *G. holbrooki* downstream of the Fenholloway being exposed to a greater load of progesterones and androgens, which could explain the increases of expression of reproductive genes [40] as well as anal fin elongation via breakdown of P4 into androgenic compounds [37].

At other paper mill sites, the phytoestrogen β-sitosterol is thought to be the causative estrogenic chemical [41], and this chemical has been found (but not quantified) at the Fenholloway River paper mill-impacted site [39]. The presence of phytoestrogens is an area of future work towards determining the causative chemicals and impacts of paper mill exposure in the Fenholloway River ecosystem. In addition, there may be synergistic interactions between non-androgenic chemicals present in the paper mill effluent, as these effluents represent complex mixtures with a diversity of effects [32] that may be causing the manifestation of abnormal secondary sexual characteristics at this site.

While AED was initially thought to be the causative masculinizing chemical, a follow-up exposure of *G. affinis* to doses of AED present in the Fenholloway River (0.04 µg/L) showed no statistically significant increase in anal fin elongation after 6 weeks of exposure [10]. These impacts were not evaluated on early life stages, which may be more sensitive to androgen exposure, but nonetheless it appears that AED may not be the masculinizing chemical in this system, and other currently unknown androgenic and progestagenic chemical(s) may be present. Additional studies which focus on the role of sediments as well as the transferability of chemicals from adult female *G. holbrooki* to their offspring during oocyte development would help address the role of soil contact and maternal transfer of paper mill exposure in this environment.

## Conclusions

Our findings indicate that a mixture of progesterones and androgens may be driving changes in gene expression at this paper mill-impacted site. These findings provide the insights into the complex nature and persistence of gene expression patterns that coincide with male phenotypic characteristics, and can serve as an initial research focus towards efforts to examine exposure routes and possible chemical sinks in field samples at a site with a population of *G. holbrooki* that has exhibited abnormal physiology for over 30 years.

## Supporting Information

File S1 **File S1 contains qPCR primer sequences (Table S1), hierarchical cluster analysis for the paper mill and reference site females (Fig. S1), PathwayStudio analysis of metabolic gene changes during paper mill exposure (Fig. S2), and standard curves for both the AR and PR GeneBLAzer assays are provided (Fig. S3).**
(DOCX)

File S2 **File S2 contains fold change levels of all transcripts that were significantly differentially regulated from the paper mill-impacted and reference sites.**
(XLSX)

File S3 **File S3 is a list of genes with similar regulation between the paper mill-impacted** ***G. holbrooki*** **and** ***G. holbrooki*** **exposed to the potent androgen receptor agonist 17β-trenbolone.**
(XLS)
