# Supplementary material for: Exposure to Paper Mill Effluent at a Site in North Central Florida Elicits Molecular-Level Changes in Gene Expression Indicative of Progesterone and Androgen Exposure
Source: PLoS One. 2014 Sep 8;9(9):e106644. doi: 10.1371/journal.pone.0106644 (PMC4157789; doi:10.1371/journal.pone.0106644)
Supplement: File S1 — contains qPCR primer sequences (Table S1), hierarchical cluster analysis for the paper mill and reference site females (Fig. S1), PathwayStudio analysis of metabolic gene changes during paper mill exposure (Fig. S2), and standard curves for both the AR and PR GeneBLAzer assays are provided (Fig. S3). (DOCX) [file pone.0106644.s001.docx]

File S1 supplemental information for:

**Exposure to paper mill effluent at a site in North Central Florida elicits molecular-level changes in gene expression indicative of progesterone and androgen exposure**

Erica K. Brockmeier, Sumith Jayasinghe, William E. Pine, Krystan A. Wilkinson, and Nancy D. Denslow

Number of pages: 6

Number of tables: 1

Number of figures: 3

| **Table S1.** qPCR primers utilized in this study | |
| --- | --- |
| Gene name | qPCR primers (5’ to 3’) |
| *17-beta hydroxysteroid dehydrogenase 3* | Forward: GCAAGGCTTACAGGACTACGTT Reverse: TCACAGCATCCATCGGTTC |
| *Ribosomal protein L8* | Forward: AACTACGCCACCGTCATCTC Reverse: CAGGATGGGCTTGTCGATAC |
| *Sonic hedgehog* | Forward: TGTCCTCATCGCTGTTCATC Reverse: AGTGGATGGATCGAGTGGTC |
| *Vitellogenin* | Forward: CAGCAGTTCCAAACGCAGTA Reverse: CTTGATCTGCCACTCCTGCT |
| *Zona pellucida glycoprotein 2* | Forward: CAGTGGGTCCTTCATCTGGT Reverse: GGGTTCCATTGAATTGGTGT |

**
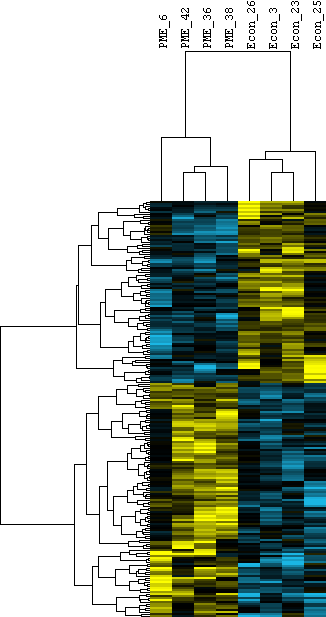
**

**Figure S1.** Gene expression differences between Fenholloway (PME) and reference site (Econ) female G. holbrooki as determined by hierarchical cluster analysis. Genes with fold changes > 1.5 fold or < -1.5 fold from the reference site and with one-way ANOVA p-values < 0.05 (false discovery rate α = 0.05) were used for this analysis. 91 genes were down-regulated at the paper mill site as compared to the reference site and 121 genes were up-regulated at the paper mill site as compared to the reference site. Data were median-centered by gene and clustered using centered correlation and complete linkage.


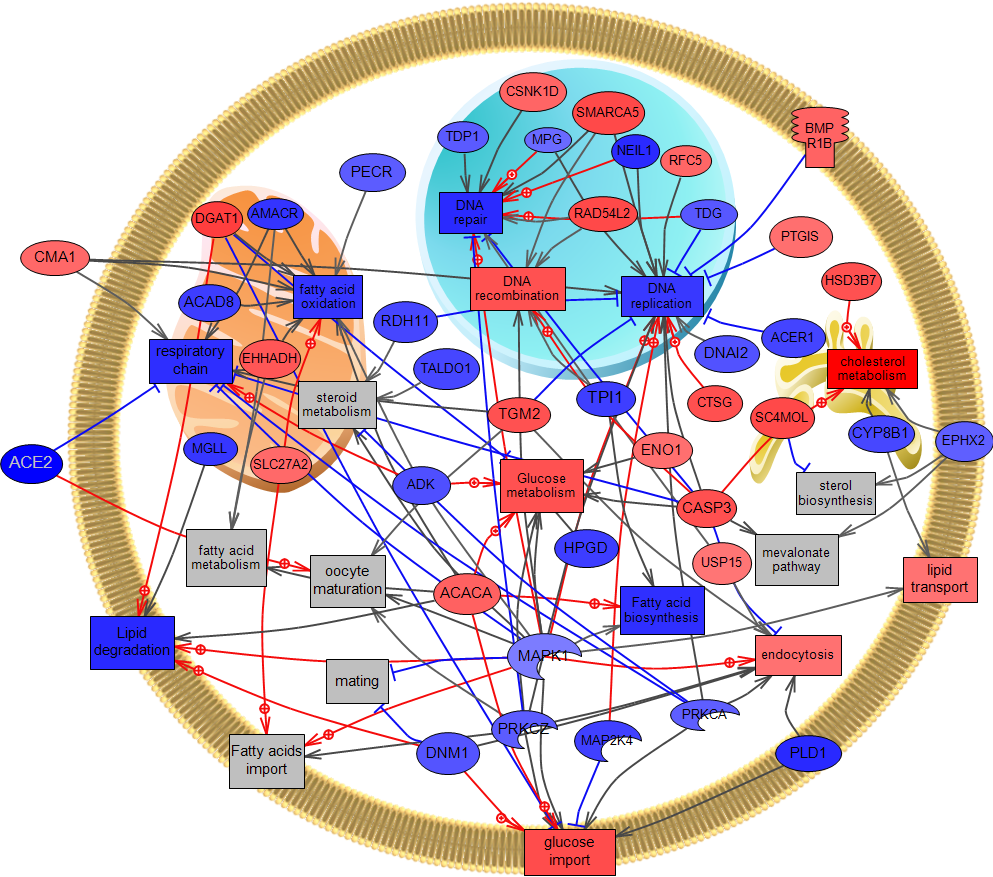


**Figure S2.** Visualization of interconnected pathways in hepatic gene expression profiles by PathwayStudio^TM^ (Elsevier, Amsterdam, The Netherlands). Enriched gene ontology Biological Processes at the Fenholloway, as determined using human homologues, and their connection to significantly up or down-regulated genes in the liver can be seen. Red genes and processes are up-regulated at the paper mill site versus the reference site whereas blue genes and processes are down-regulated. Red lines are positive gene regulation, blue lines are negative regulation, and grey lines ending in arrows have an unknown direction of regulation. Boxes represent biological processes and ovals represent individual genes, and the intensity of the coloring is indicative of the intensity of expression differences. Gene abbreviations: ACACA (acetyl coenzyme A carboxylase alpha), ACAD8 (acyl-CoA dehydrogenase family, member 8), ACE2 (angiotensin I converting enzyme 2), ACER1 (alkaline ceramidase 1), ADK (adenosine kinase), AMACR (alpha-methylacyl-CoA racemase), BMPR1B (bone morphogenetic protein receptor, type IB), CASP3 (caspase 3), CMA1 (chymase 1), CSNK1D (casein kinase 1, delta), CTSG (cathepsin G), CYP8B1 (cytochrome P450 family 8, subfamily B, polypeptide 1), DGAT1 (diacylglycerol O-acyltransferase homolog 1), DNAI2 (dynein, axonemal intermediate chain 2), DNM1 (dynamin 1), ENO1 (enolase 1), EHHADH (enoyl-coA, hydratase/3-hydroxyacyl CoA dehydrogenase), EPHX2 (epoxide hydrolase 2), HPGD (hydroxyprostaglandin dehydrogenase 15-(NAD)), HSD3B7 (hydroxyl-delta-5-steroid dehydrogenase, 3-beta and steroid delta-isomerase 7), MAPK1 (mitogen-activated protein kinase 1), MAP2K4 (mitogen-activated protein kinase 4), MGLL (monoglyceride lipase), MPG (N-methylpurine-DNA glycosylase), NEIL1 (nei endonuclease VIII-like), PECR (peroxisomal trans-2-enoyl-CoA reductase), PLD1 (phospholipase D), PRKCA (protein kinase C alpha), PTGIS (prostaglandin I2 (prostacyclin) synthase), RAD54L2 (Androgen receptor-interacting protein 4), RDH11 (retinol dehydrogenase 11), RFC5 (replication factor C), SC4MOL (sterol-C4-methyl oxidase-like), SLC27A2 (solute carrier family 27, fatty acid transporter, member 2), SMARCA5 (SWI/SNF related, matrix associated, actin dependent regulator of chromatin, subfamily A, member 5), TALDO1 (transaldolase 1), TDG (thymine-DNA glycosylase), TDP1 (tyrosyl-DNA phosphodiesterase 1), TGM2 (transglutaminase 2), TPI1 (triosephosphate isomerase 1), USP15 (ubiquitin specific peptidase 15).

***
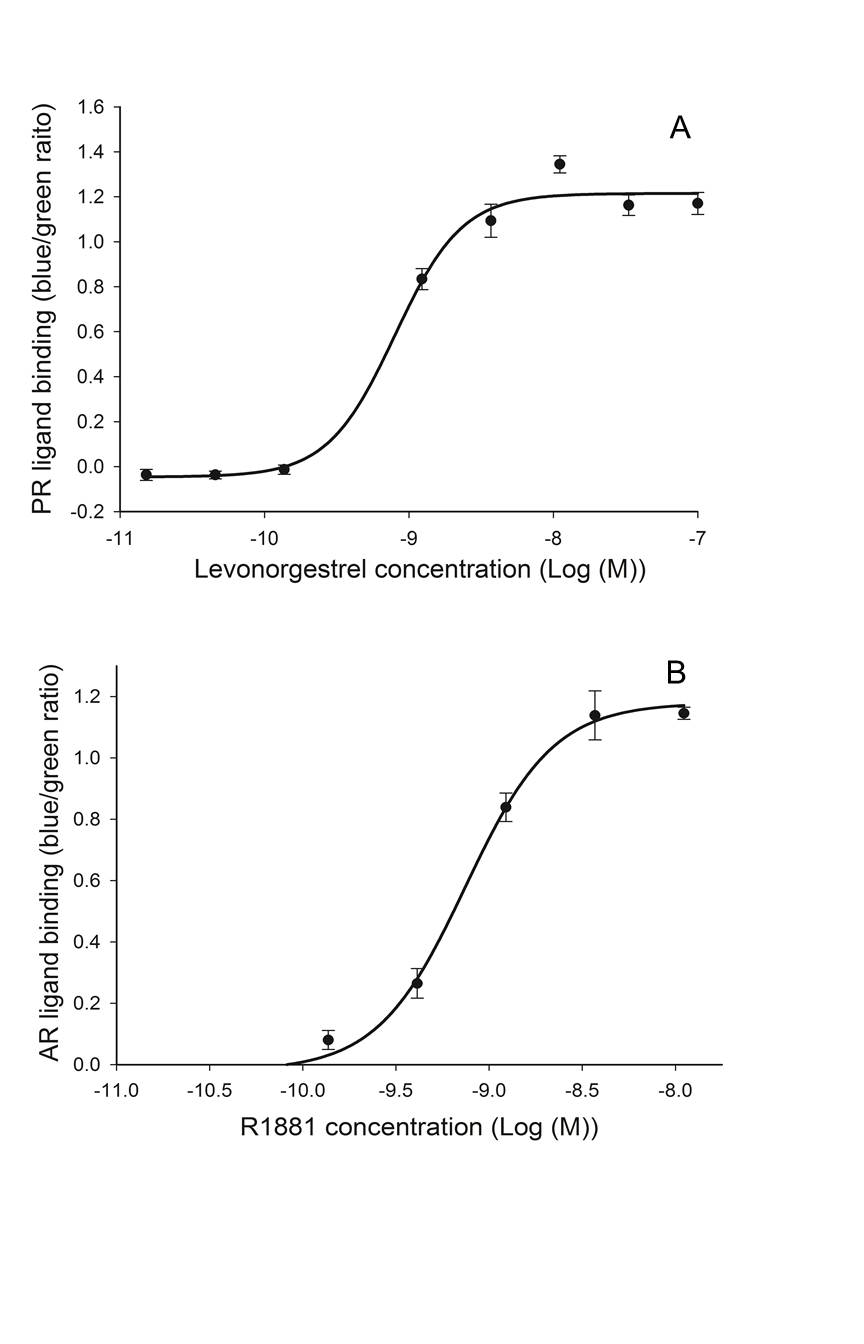
***

**Figure S3.** Standard curves of levonorgestrel and R1881 for the progesterone receptor (A) and androgen receptor (B) Gene BLAzer assays. Each dot represents the average of three replicates from each concentration and error bars are standard deviation of the three replicates.
